# Adsorbing the magnetic superhalogen MnCl_3_ to realize intriguing half-metallic and spin-gapless-semiconducting behavior in zigzag or armchair SiC nanoribbon†

**DOI:** 10.1039/c8ra01632a

**Published:** 2018-04-11

**Authors:** Hui Li, Guangtao Yu, Zengsong Zhang, Yanfeng Ma, Xuri Huang, Wei Chen

**Affiliations:** Laboratory of Theoretical and Computational Chemistry, Institute of Theoretical Chemistry, International Joint Research Laboratory of Nano-Micro Architecture Chemistry, Jilin University Changchun 130023 People’s Republic of China yugt@jlu.edu.cn w_chen@jlu.edu.cn

## Abstract

By means of first-principles computations, we first propose a new and effective strategy through adsorbing the magnetic superhalogen MnCl_3_ to modulate the electronic and magnetic properties of zigzag- and armchair-edged SiC nanoribbons (zSiCNR and aSiCNR, respectively). In view of its large intrinsic magnetic moment and strong electron-withdrawing ability, the adsorption of magnetic superhalogen MnCl_3_ can introduce magnetism in the substrate SiCNR, and simultaneously induce the electron transfer process from SiCNR to MnCl_3_, resulting in the evident increase of electrostatic potential in the ribbon plane, like applying an electric field. As a result, the magnetic degeneracy of pristine zSiCNR can be broken and a robust ferromagnetic half-metallicity or metallicity can be observed in the modified zSiCNR systems, while a robust ferromagnetic half-metallic or spin-gapless-semiconducting behavior can be obtained in the modified aSiCNR systems. Note that both the appealing half-metallicity and spin-gapless-semiconductor behavior are key features which hold promise for future spintronic applications. Moreover, all of these new superhalogen–SiC nanosystems can possess considerably high structural stabilities. These intriguing findings will be advantageous for promoting excellent SiC-based nanomaterials in the applications of spintronics and multifunctional nanodevices in the near future.

## Abstract 2

Adsorbing a magnetic superhalogen can be an effective strategy to achieve intriguing half-metallicity and spin-gapless-semiconductor behavior in SiC nanoribbon.

## Graphical abstract

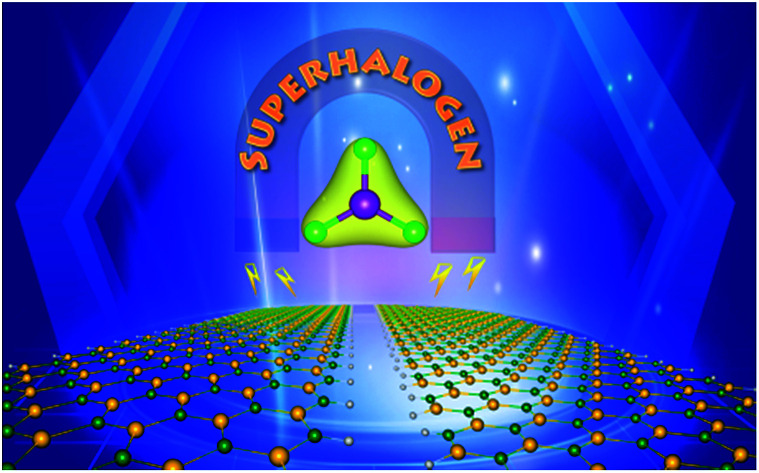

## 1 Introduction

The discovery of isolated graphene, an extended honeycomb network of sp^2^-hybridized carbon atoms, has totally refreshed our minds and opened the gate to low dimensional nanomaterials.^1,2^ Owing to its reduced dimensions, graphene can possess many fascinating physical properties,^3–5^ such as massless Dirac Fermion behavior,^3^ high mobility,^4^ and the largest strength measured so far.^5^ Inspired by these captivating properties, great effort has been made not only for graphene-based materials, but also for analogous inorganic materials.^6–18^

The inorganic SiC nanoribbon (SiCNR), as a structural analogue to graphene nanoribbon (GNR), has become a rising star in the family of inorganic nanomaterials, and is attracting great interest from many experimental and theoretical research groups. It is well known that SiC as a leading material can be extensively applied in harsh environments (*e.g.* high temperature, pressure or power),^19–22^ since it possesses numerous outstanding characteristics, such as a large mechanical strength, high thermal conductivity, as well as excellent resistance to oxidation and corrosion.^23–26^ Experimentally, different polymorphs of SiC nanoribbons have been synthesized *via* several routes.^27–31^ For example, *via* a catalyst-free route at a relatively low growth temperature, wurtzite-type SiC (2H–SiC) nanoribbons have been fabricated,^27^ which are tens to hundreds of microns in length, a few microns in width and tens of nanometers in thickness. By a lithium-assisted synthetic route or the thermal evaporation approach, SiC (3C–SiC) nanoribbons have been obtained.^28^ In addition, through the nanosecond pulsed laser direct-write and doping (LDWD) technique, carbon-rich SiC nanoribbons have been fabricated, which are proposed as transistor–resistor interconnects for nanodevices and photonic band-gap arrays in microstrip circuits.^29^

Besides, considerable theoretical work has been focused on one-dimensional (1D) inorganic SiCNRs with a zigzag or armchair edge. By means of first principles calculations, the ground state of zigzag SiCNR (zSiCNR) is predicted to be energetically degenerate with ferromagnetic (FM) and antiferromagnetic (AFM) configurations, where the metallic and half-metallic behaviors can be observed, respectively.^32^ However, due to the magnetic degeneracy, the FM metallicity and AFM half-metallicity in pristine zSiCNR are vulnerable to even small disturbances, inhibiting its practical application in spintronics and multifunctional nanodevices. Comparatively, armchair SiCNR (aSiCNR) can exhibit nonmagnetic semiconducting behavior with a band gap of about 2.373 eV,^33,34^ and such a large band gap is also not advantageous for its application in functional nanodevices.^35,36^ To conquer these bottlenecks, some approaches have been proposed to modulate the electronic and magnetic properties of zSiCNR and aSiCNR systems,^37–43^ for example, hydrogenation,^37^ applying an electric field,^38,39^ edge modification with functional groups/atoms,^40,41^ and (non)covalent surface modification with an appropriate molecule/polymer.^42,43^

Differing from previously reported approaches, in this work we propose a new and effective strategy through adsorbing a magnetic superhalogen to tune the electronic and magnetic properties of zigzag- and armchair-edged SiC nanoribbons. It is well known that a superatom is a stable assembly of atoms that mimics the behavior of elemental atoms, and could serve as potential building blocks for the assembly of new materials with desired properties.^44–52^ As one of the most important members in the family of superatoms, the superhalogens can possess larger electron affinities (EAs) than the halogen atoms, and have been extensively used in the design of novel materials.^50–52^ Mn_*x*_Cl_*y*_ can be considered as a unique moiety of the superhalogen group, and has been recently studied theoretically and synthesized experimentally.^53,54^ Here, the magnetic superhalogen MnCl_3_ has been sampled to modulate the electronic and magnetic behaviors of SiCNR. We can understand that neutral MnCl_3_ possesses a considerable spin magnetic moment (*ca.* 4.00 *μ*_B_) and a large electron affinity (*ca.* 4.94 eV), even larger than that of the Cl atom (3.62 eV).^55^ Thus, we can reasonably speculate that adsorbing MnCl_3_ could induce an evident change in electrostatic potential and simultaneously introduce magnetism to the substrate SiCNR, in view of the strong electron-withdrawing ability and intrinsic magnetic moment. It is highly anticipated that the adsorption of the magnetic superhalogen MnCl_3_ can effectively engineer the band structure of SiCNR, and so obtain captivating electronic and magnetic properties such as half-metallicity^40^ and spin gapless semiconductor (SGS),^37,56^ both of which are key features which hold promise for future spintronics applications.

In this study, we have carried out systematic density functional theory computations to investigate the structures, and the electronic and magnetic properties of zigzag- and armchair-edged SiCNR systems by adsorbing the magnetic superhalogen MnCl_3_. We will mainly address the following issues: (1) can the adsorption of MnCl_3_ effectively tune the electronic and magnetic properties of zSiCNR and aSiCNR, and can the intriguing half-metallic or SGS behaviors be obtained? (2) How will the electronic and magnetic properties of modified zSiCNR or aSiCNR systems be affected when moving MnCl_3_ from the ribbon center to the edge? (3) Can the ribbon width have an impact on the electronic and magnetic behaviors of modified SiCNR systems? (4) How about the adsorption energies when depositing the magnetic superhalogen MnCl_3_ on the surface of SiCNRs? Undoubtedly, resolving these interesting issues will be advantageous for promoting the practical applications of excellent SiC-based nanomaterials in spintronics and multifunctional nanodevices.

## 2 Computational methods

All calculations are performed within the framework of density functional theory (DFT) as implemented in the Vienna *Ab initio* Simulation Package (VASP).^57–60^ The electron–ion interaction is described by a projector augmented wave (PAW)^61^ method using Perdew–Burke–Ernzerhof (PBE)^62^ with van der Waals (vdw) correction proposed by Grimme (DFT-D2).^63^ Vacuum regions of 15 Å along the non-periodic directions are used to avoid interaction of the periodic images. For the modified SiCNR systems with one MnCl_3_ in the supercell, there are five and three repeated units along the ribbon length for the corresponding substrates zSiCNR and aSiCNR. Moreover, a plane wave basis with a kinetic energy cutoff of 500 eV is used, which is found to yield well-converged total energies. 1 × 1 × 11 Monkhorst–Pack grid *k*-points are employed for the geometric relaxation, and the total energy convergence threshold is set as 10^−4^ eV. On the basis of equilibrium structures, 21 *k*-points between every two high symmetry *k*-points along the one-dimensional Brillouin zone are used to obtain the band structures.^64^ The amount of electron transfer between the adsorbed MnCl_3_ and SiC nanoribbon is estimated from the Bader charge analysis.

The adsorption energy of MnCl_3_ on the surface of SiCNR is calculated according to the following expression:*E*_ad_ = *E*_SiCNR+MnCl_3__ – (*E*_SiCNR_ + *E*_MnCl_3__)where *E*_SiCNRs+MnCl_3__ is the total energy of the modified SiCNR system with MnCl_3_, *E*_SiCNR_ means the energy of pristine SiCNR, and *E*_MnCl_3__ is the energy of single MnCl_3_. The negative value of *E*_ad_ reflects the exothermic (energetically favorable) process for adsorbing MnCl_3_ on the surface of SiCNR. The large absolute value of *E*_ad_ means there is a strong interaction between MnCl_3_ and SiCNR.

## 3 Results and discussion

### 3.1 The electronic and magnetic properties of pristine SiC nanoribbons

**Fig. 1:**
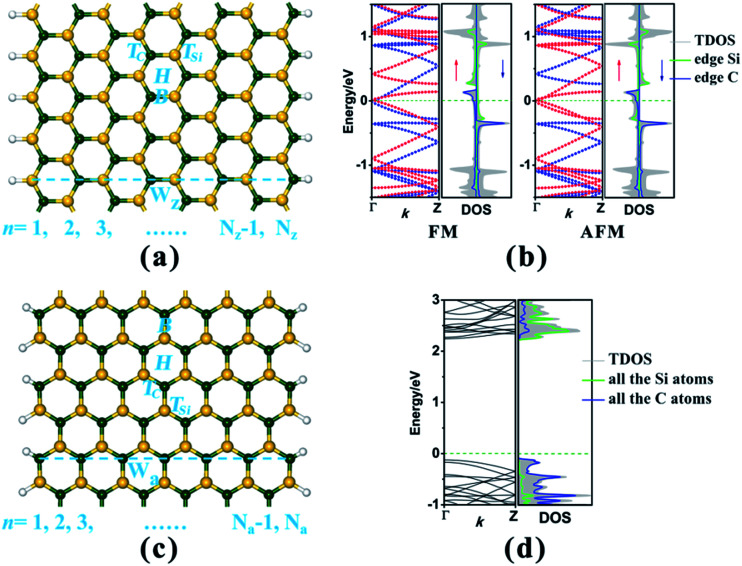
(a) The geometrical structure of H-terminated 8-zSiCNR. (b) The electronic band structures and corresponding DOS plots of pristine 8-zSiCNR in FM and AFM states, in which the red and blue dotted lines in the band structures represent the spin-up (↑) and spin-down (↓) channels, respectively. (c) The geometrical structure of H-terminated 13-aSiCNR. (d) The electronic band structure and corresponding DOS plot of pristine 13-aSiCNR in the NM state. The Fermi-level is set as zero and is indicated by a green dotted line. The yellow, dark green and white balls in the geometrical structures represent Si, C and H atoms, respectively.

In this study, we have considered two types of SiC nanoribbon with different edge chiralities, namely zigzag SiCNR (zSiCNR) and armchair SiCNR (aSiCNR), as illustrated in Fig. 1a and c. According to convention, different widths of zSiCNRs and aSiCNRs can be denoted as *N*_z_-zSiCNR and *N*_a_-aSiCNR, respectively, where *N*_z_ or *N*_a_ is the number of parallel zigzag chains or dimer lines across the corresponding ribbon width (Fig. 1a and c). All of the studied SiCNRs are terminated by hydrogen atoms to passivate the dangling bond on the ribbon edge. We take 8-zSiCNR and 13-aSiCNR as prototype systems and investigate the effect of adsorbing the magnetic superhalogen MnCl_3_ upon the electronic and magnetic properties of zigzag- and armchair-edged SiCNR systems.

To make a comparison, we initially investigate the geometrical structures, and the electronic and magnetic properties of pristine 8-zSiCNR and 13-aSiCNR. Both spin-polarized and spin-unpolarized computations have been performed, where the ferromagnetic (FM), antiferromagnetic (AFM) and nonmagnetic (NM) states are considered. Our computed results reveal that for pristine 8-zSiCNR, the unpaired spin is mainly concentrated on the edge Si and C atoms, and their orientations are parallel and antiparallel between the Si and C edges for FM and AFM states respectively. Both the states are energetically degenerate, and yet much lower than the corresponding NM state, indicating that the degenerate FM and AFM configurations are the ground state of pristine zSiCNR. Furthermore, our computed band structures show that the pristine zSiCNR can exhibit metallic behavior in the FM state and half-metallic behavior in the AFM state. The computed results on the density of states (DOS) reveal that the FM metallicity can be dominated by spin-up and spin-down states across the Fermi-level mainly originating from the edge C and edge Si atoms, respectively, while the AFM half-metallicity can be determined by the spin-up states from the edge C and Si atoms crossing the Fermi-level (Fig. 1b). Comparatively, our computed results reveal that pristine 13-aSiCNR is a nonmagnetic semiconductor with a direct band gap as large as 2.367 eV, where the top valence band (TVB) and bottom conduction band (BCB) are from the C and Si atoms, respectively (Fig. 1d). It is worth mentioning that all these computed results on pristine zSiCNR and aSiCNR are consistent with earlier reports.^32–34^

### 3.2 The geometrical structures, and electronic and magnetic properties of SiCNRs with the adsorbed MnCl_3_ at the center

In this section we have performed a detailed investigation to explore the effect of adsorbing the magnetic superhalogen MnCl_3_ at the ribbon center upon the geometrical structures, and the electronic and magnetic properties of zigzag- and armchair-edged SiCNR systems, by sampling the corresponding 8-zSiCNR and 13-aSiCNR. Our computed results reveal that the sole MnCl_3_ possesses a large electron affinity (*ca.* 4.728 eV) and spin magnetic moment (*ca.* 4.00 *μ*_B_), consistent with those reported previously.^53^ Consequently, adsorbing the magnetic superhalogen MnCl_3_, with its strong electron-withdrawing ability as well as its intrinsic magnetic moment, could significantly change the electrostatic potential and simultaneously introduce magnetism to the substrate SiCNR. It is highly anticipated that this kind of surface modification by the appealing magnetic superhalogen unit can effectively modulate the electronic and magnetic behaviors for zigzag- and armchair-edged SiC nanoribbons, promoting the practical application of SiC-based nanomaterials in multifunctional and spintronic nanodevices.

Initially, we focus on the adsorption of MnCl_3_ at the ribbon center for 8-zSiCNR and 13-aSiCNR, where all four possible adsorption sites for the Mn atom are considered, including the top site of the C atom (T_C_), the top site of the Si atom (T_Si_), the bridge site over the Si–C bond (B) and the hollow site of the SiC hexagonal ring (H), as illustrated Fig. 1a and c. Note that these MnCl_3_-modified 8-zSiCNR and 13-aSiCNR systems are named MnCl_3_-8-zSiCNR-center and MnCl_3_-13-aSiCNR-center, respectively, where “center” denotes that the adsorption site of MnCl_3_ is at the ribbon center. Our computed results reveal that MnCl_3_-T_C_-8-zSiCNR-center and MnCl_3_-T_C_-13-aSiCNR-center are the most stable among their respective possible zSiCNR or aSiCNR configurations with the adsorption of MnCl_3_ at the center, as discussed in detail in the ESI (Fig. S1 and S2†).

In these two stable MnCl_3_-T_C_-8-zSiCNR-center and MnCl_3_-T_C_-13-aSiCNR-center configurations, the original planar structure of MnCl_3_ can be deformed owing to the formation of a Mn–C bond (2.281 Å for the former and 2.107 Å for the latter). The computed adsorption energies are −2.319 eV and −1.430 eV respectively (Table 1), and such the large negative adsorption energy suggests that adsorbing the magnetic superhalogen MnCl_3_ on the surface of SiCNR can be an energetically favorable process, and that the resulting joint system can possess high structural stability.

**Table 1:** The relative energies Δ*E* (meV) of different magnetic couplings to the ground state, the total magnetic moment *M*_tot_, the distance *d*_Mn–C_ between the Mn atom in the adsorbed MnCl_3_ and the bonded C atom, the adsorption energy *E*_ad_, the electronic property and the amount of electron transfer from SiCNR to MnCl_3_ for MnCl_3_-T_C_-8-zSiCNR-center and MnCl_3_-T_C_-13-aSiCNR-center

Systems	Δ*E* (meV)				*M*_tot_ (*μ*_B_)	*d*_Mn–C_ (Å)	*E*_ad_ (eV)	Electronic property	The charge of the SiCNR|*e*|
	NM	FM	FM-1^a^	AFM					
8-zSiNR	166.7	0.0	—	0.0	2.14/0.0	—	—	—	—
MnCl_3_-T_C_-8-zSiCNR-center	—	0.0	31.6	—	7.10	2.281	−2.319	Metallicity	0.905
13-aSiCNR	0.0	—	—	—	—	—	—	Semiconductor^b^	—
MnCl_3_-T_C_-13-aSiCNR-center	1.798	0.0	—	—	4.00	2.107	−1.430	Half-metallicity^c^	0.681

a FM-1 represents the parallel coupling between MnCl_3_ and the C-edge, accompanied by the antiparallel coupling between the Si and C edges.

b The band gap is 2.367 eV.

c The gap in the minority channel is 1.463 eV.

**Fig. 2:**
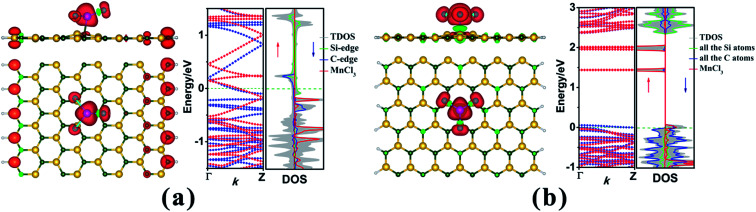
The geometry, electron spin density distribution, band structure and corresponding DOS for the most favorable SiCNR-based configurations with adsorbed MnCl_3_ at the ribbon center, namely, (a) MnCl_3_-T_C_-8-zSiCNR-center and (b) MnCl_3_-T_C_-13-aSiCNR-center. The red and green colors in the electron spin density distribution represent the spin-up and spin-down orientations of the unpaired electrons, respectively. The red and blue dotted lines in the band structures denote the spin-up (↑) and spin-down (↓) channels, respectively. The Fermi-level is set as zero and is indicated by the green dotted line.

Furthermore, we explore the effect of adsorbing MnCl_3_ upon the electronic and magnetic properties of pristine zigzag- and armchair-edged SiCNRs. The computed results reveal that the adsorption of MnCl_3_ over the ribbon center can break the magnetic degeneracy of pristine zSiCNR, and that sole FM metallicity can be observed in MnCl_3_-T_C_-8-zSiCNR-center (Fig. 2a and Table 1). The corresponding total magnetic moment *M*_tot_ is as large as 7.10 *μ*_B_ per supercell. Clearly, this approach can overcome the problem of FM metallicity and AFM half-metallicity being vulnerable to even small disturbances owing to the energy degeneracy of FM and AFM states for pristine zSiCNR, which is advantageous for promoting practical application in multifunctional nanodevices. The computed DOS results show that the metallicity in MnCl_3_-T_C_-8-zSiCNR-center is mainly determined by the spin-up and spin-down states from the respective edge C and edge Si atoms across the Fermi level (Fig. 2a). Comparatively, adsorbing MnCl_3_ over the ribbon center of 13-aSiCNR can transform the original NM semiconductor such that it exhibits intriguing FM half-metallicity (Fig. 2b and Table 1), where the total magnetic moment *M*_tot_ is as large as 4.00 *μ*_B_ per supercell and the gap in the minority channel is as large as 1.463 eV. Such a large semiconducting spin gap suggests that the half-metallicity is rather robust and there is great potential for experimental realization.^65^ Note that great effort has been made in exploring the half-metallicity in low-dimensional nanomaterials (*e.g.*, GNRs^10,14^ and BNNRs^66,67^), in view of their potential application in spintronics. Moreover, the computed DOS results reveal that the half-metallic behavior in MnCl_3_-T_C_-13-aSiCNR-center can be mainly dominated by the spin-down state crossing the Fermi level from MnCl_3_ and C atoms (Fig. 2b).

**Fig. 3:**
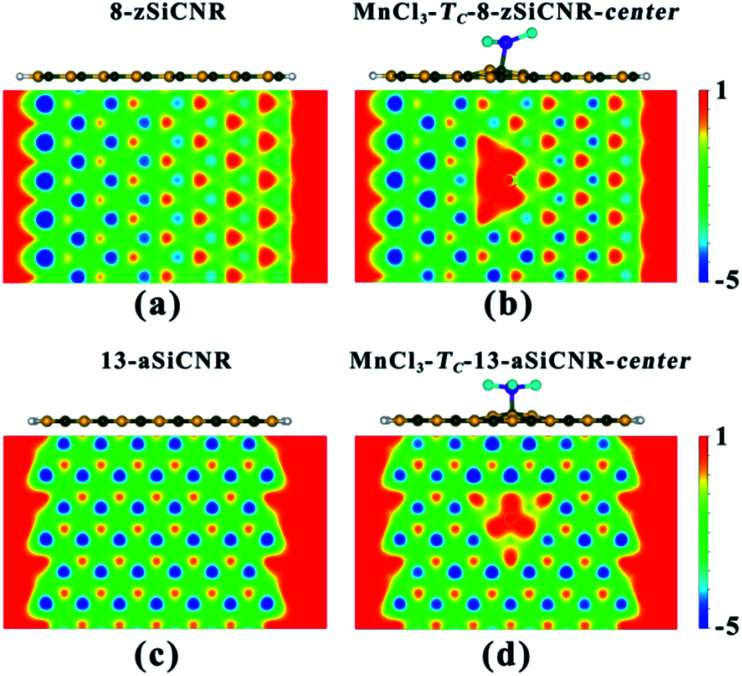
The side view of the geometrical structure and the distribution of electrostatic potential in the nanoribbon plane for the pristine SiCNRs and the corresponding MnCl_3_-modified SiCNR systems at the center: (a) pristine 8-zSiCNR, (b) MnCl_3_-T_C_-8-zSiCNR-center, (c) pristine 13-aSiCNR and (d) MnCl_3_-T_C_-13-aSiCNR-center.

To understand the reason why adsorbing superhalogen MnCl_3_ can effectively engineer the band structures of pristine zSiCNR and aSiCNR, we have performed computations on the electrostatic potential and Bader charge for the two most stable conformations, namely, MnCl_3_-T_C_-8-zSiCNR-center and MnCl_3_-T_C_-13-aSiCNR-center. As illustrated in Fig. 3, independent of the edge chirality, the substrate SiCNR in the modified system can possess a different distribution of electrostatic potential from that of pristine SiCNR, where the adsorption of strong electron-withdrawing MnCl_3_ can induce an increase of electrostatic potential in the substrate of SiCNR. This situation can be due to the occurrence of electron transfer from the substrate (*ca.* 0.905|*e*| and 0.681|*e*| for 8-zSiCNR and 13-aSiCNR, respectively) to MnCl_3_ (Table 1). Obviously, the adsorption of superhalogen MnCl_3_ can result in an evident change of electrostatic potential in SiCNR, just like applying an electric field, which is mainly responsible for engineering the band structures of zigzag- and armchair-edged SiCNRs. It is worth mentioning that when applying an external electric field, the magnetic degeneracy of zSiCNR can be broken and that sole FM metallic behavior can be observed,^38^ while the band gap of aSiCNR can be decreased with an increase of field strength and even metallic behavior can be obtained.^39^

Clearly, adsorbing the magnetic superhalogen MnCl_3_ at the ribbon center can effectively modulate the electronic and magnetic behaviors of SiCNR with the zigzag or armchair edge. The magnetic degeneracy of pristine zSiCNR can be broken and sole FM metallicity can be achieved, while the NM semiconductor of aSiCNR can be changed so as to exhibit a robust FM half-metallicity. These intriguing findings will be advantageous for promoting the practical application of SiC-based nanomaterials in multifunctional and spintronic nanodevices.

### 3.3 The geometrical structures, and the electronic and magnetic properties of SiCNRs with the adsorbed MnCl_3_ at the ribbon edge

Based on the above discussion, we can understand that adsorbing the magnetic superhalogen MnCl_3_ over the ribbon center can effectively modulate the electronic and magnetic properties of SiC nanoribbons, such that FM metallicity and FM half-metallicity can be observed in the modified zSiCNR and aSiCNR systems, respectively. In this section, we will explore the effect of moving MnCl_3_, from the ribbon center to the edge, on the electronic and magnetic properties of zigzag- and armchair-edged SiCNRs. It is well known that nanoribbon edges are more chemically active, thus it is highly anticipated that adsorbing MnCl_3_ at the edge can also effectively engineer the band structures of SiCNRs and even induce intriguing electronic and magnetic properties such as half-metallic and SGS behaviors, both of which are key features which hold promise for future spintronic applications.

**Fig. 4:**
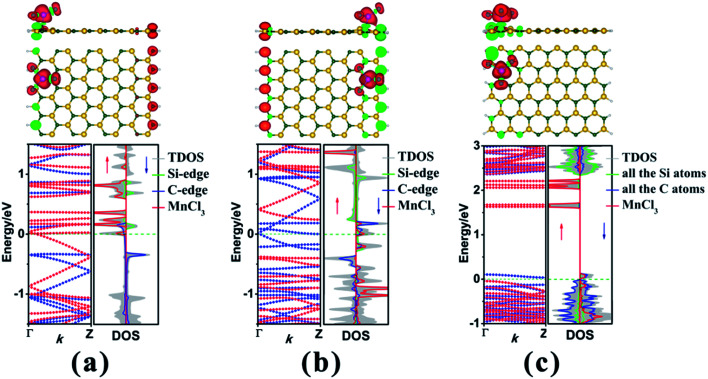
The geometry, electron spin density distribution, band structure and corresponding DOS for the MnCl_3_-modified SiCNR systems at the ribbon edge, namely, (a) MnCl_3_-T_C_-8-zSiCNR-eSi, (b) MnCl_3_-T_C_-8-zSiCNR-eC and (c) MnCl_3_-T_C_-13-aSiCNR-edge. The red and green colors in the electron spin density distribution represent the spin-up and spin-down orientations of the unpaired electrons, respectively. The red and blue dotted lines in the band structures denote the spin-up (↑) and spin-down (↓) channels, respectively. The Fermi-level is set as zero and is indicated by the green dotted line.

Initially, we take the most stable MnCl_3_-T_C_-8-zSiCNR-center and MnCl_3_-T_C_-13-aSiCNR-center systems with MnCl_3_ adsorbed at the center as the starting points to examine the effect of the adsorption site upon the electronic and magnetic properties of zSiCNR and aSiCNR by moving MnCl_3_ from the ribbon center to the edge, where the Mn atom is still located atop the C atom. As shown in Fig. 4a and b, while changing the position of MnCl_3_ towards the Si/C edge of zSiCNR, two corresponding conformations MnCl_3_-T_C_-8-zSiCNR-eSi and MnCl_3_-T_C_-8-zSiCNR-eC can be obtained, where “eSi and “eC” mean the adsorption site of MnCl_3_ located at the Si edge and C edge, respectively. Similarly, when moving MnCl_3_ to the edge of aSiCNR, we can obtain one new configuration MnCl_3_-T_C_-13-aSiCNR-edge (Fig. 4c), where the “edge” means the adsorption site located at the edge of aSiCNR. Our computed results reveal that the distances between the Mn atom and the bonded C atom are 2.260, 2.175 and 2.179 Å for MnCl_3_-T_C_-8-zSiCNR-eSi, MnCl_3_-T_C_-8-zSiCNR-eC and MnCl_3_-T_C_-13-aSiCNR-edge, respectively (Table 2). All of these three configurations with MnCl_3_ at the ribbon edge can possess considerable adsorption energies of −3.865, −1.826 and −1.719 eV, respectively (Table 2). Particularly, the MnCl_3_-modified zSiCNR at the Si edge and MnCl_3_-modified aSiCNR at the ribbon edge can even possess larger absolute values than the corresponding ones with MnCl_3_ at the center, indicating that both of them are more stable in energy.

**Table 2:** The relative energies Δ*E* (meV) of different magnetic couplings to the ground state, the total magnetic moment *M*_tot_, the distance *d*_Mn–C_ between the Mn atom in the adsorbed MnCl_3_ and the bonded C atom, the adsorption energy *E*_ad_, the electronic property and the amount of the electron transfer from SiCNR to MnCl_3_ for the modified zSiCNR and aSiCNR systems with the MnCl_3_ at the ribbon edge

Systems	Δ*E* (meV)			*M*_tot_ (*μ*_B_)	*d*_Mn–C_ (Å)	*E*_ad_ (eV)	Electronic property	The gap in the minority channel (eV)	The charge of the SiCNR|*e*|
	NM	FM	FM-1						
MnCl_3_-T_C_-8-zSiCNR-eSi	—	5.1	0.0	6.00	2.260	−3.865	Half-metallicity	0.450	0.888
MnCl_3_-T_C_-8-zSiCNR-eC	—	139.5	0.0	4.00	2.175	−1.826	Half-metallicity	0.622	0.755
MnCl_3_-T_C_-13-aSiCNR-edge	1.939	0.0	—	4.00	2.179	−1.719	SGS	—	0.723

Subsequently, we explore the effect of the adsorption site upon the electronic and magnetic properties of these three MnCl_3_-modified zSiCNR and aSiCNR systems by computing the band structures and DOSs, including MnCl_3_-T_C_-8-zSiCNR-eSi, MnCl_3_-T_C_-8-zSiCNR-eC and MnCl_3_-T_C_-13-aSiCNR-edge. The computed results reveal that when changing the adsorption site of MnCl_3_ from the center to the Si edge of zSiCNR, the original metallicity can be transformed into half-metallicity (Fig. 4a and Table 2). Additionally, compared with the FM state (with the uniform parallel coupling between the MnCl_3_, Si and C edges) of MnCl_3_-T_C_-8-zSiCNR-center, MnCl_3_-T_C_-8-zSiCNR-eSi can also exhibit a large total magnetic moment (*M*_tot_ = 6.00 *μ*_B_), where the spin distribution is parallel between MnCl_3_ and the C edge, but antiparallel between the Si and C edges. Note that this kind of spin distribution can be also considered as the ferromagnetic state (denoted by FM-1), in view of the case that the total magnetic moment of the composite system is large, which can be mainly dominated by adsorbed MnCl_3_.

**Fig. 5:**
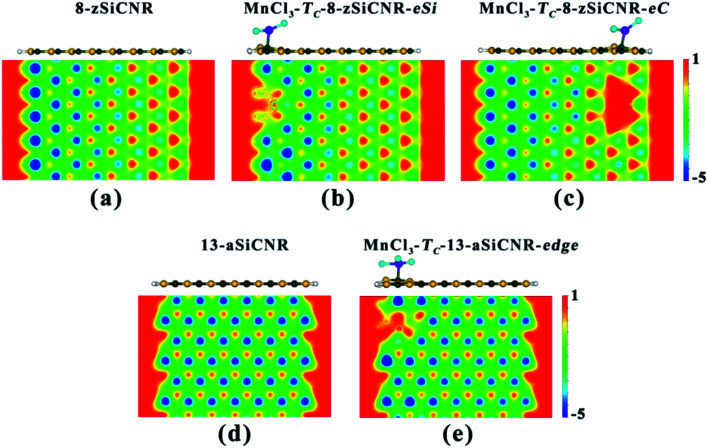
The side view of the geometrical structure and the distribution of electrostatic potential in the nanoribbon plane for the pristine SiCNRs and the corresponding MnCl_3_-modified SiCNR systems at the edge: (a) pristine 8-zSiCNR, (b) MnCl_3_-T_C_-8-zSiCNR-eSi, (c) MnCl_3_-T_C_-8-zSiCNR-eC, (d) pristine 13-aSiCNR and (e) MnCl_3_-T_C_-13-aSiCNR-edge.

Similarly, when moving MnCl_3_ from the center to the C edge of zSiCNR, a conversion of the metallicity to half-metallcity can be still achieved where the ferromagnetic state (FM-1) with a large total magnetic moment (*M*_tot_ = 4.00 *μ*_B_) is also observed (Fig. 4b and Table 2). The computed DOS results reveal that the half-metallic behaviors in MnCl_3_-T_C_-8-zSiCNR-eSi and MnCl_3_-T_C_-8-zSiCNR-eC can be dominated by the spin-up states and spin-down states crossing the Fermi level, respectively, both of which uniformly originate from the edge Si/C atoms and MnCl_3_, as illustrated in Fig. 4a and b. Clearly, adsorbing the magnetic superhalogen MnCl_3_ at the Si/C edge can also break the magnetic degeneracy of zSiCNR, owing to the evident electron transfer (*ca.* 0.888 and 0.755|*e*|, respectively) from zSiCNR to MnCl_3_ leading to an increase of electrostatic potential in the nanoribbon plane (Table 2 and Fig. 5). The robust half-metallic behavior can be uniformly observed in the two modified cases, where the semiconducting spin gaps of the minority channel are considerably large (0.450 and 0.622 eV, respectively), as shown in Table 2 and Fig. 4.

Comparatively, when moving MnCl_3_ from the center to the edge of 13-aSiCNR, the FM ground state (*M*_tot_ = 4.00 *μ*_B_) can be maintained, yet the original half-metallicity can be converted into an intriguing SGS behavior, where both the top valence band from the C atoms and the bottom conduction band from the C atoms and MnCl_3_ are in contact with each other at the Fermi-level, as illuminated in Fig. 4c. Evidently, adsorbing the superhalogen MnCl_3_ at the edge can effectively engineer the band structure of aSiCNR, due to the obvious electron transfer (*ca.* 0.723|*e*|) from aSiCNR to MnCl_3_ leading to an increase of electrostatic potential in the nanoribbon plane (Table 2 and Fig. 5e).

Overall, adsorbing the magnetic superhalogen MnCl_3_ at the edge can also effectively tune the electronic and magnetic properties of zigzag- and armchair-edged SiCNRs, in which steady ferromagnetic half-metallic or SGS behaviors can be achieved. This can be advantageous for promoting the practical application of SiC-based nanomaterials in spintronics.

### 3.4 The geometrical structures, and the electronic and magnetic properties of MnCl_3_-modified SiCNR systems with different widths

It is well known that the half-metallic and SGS behaviors in low-dimensional nanostructures have attracted great attention from researchers,^37,40,56^ owing to their promising application in spintronics. From the above discussion, we can understand that half-metallic and SGS behaviors can be observed in MnCl_3_-modified 8-zSiCNR and 13-aSiCNR systems at the ribbon edge, respectively. In this section, we ask whether both intriguing characteristics can be sustained when changing the ribbon width of correlative composite systems. We examined the effect of ribbon width (*N*_z_ or *N*_a_) upon the electronic and magnetic properties of the representative MnCl_3_-modified zSiCNR system at the Si edge (more favorable in energy) and the MnCl_3_-modified aSiCNR system at the ribbon edge by sampling *N*_z_ = 6, 10, 12 and *N*_a_ = 9, 11, 15, respectively, where the adsorption site of MnCl_3_ is kept atop the C atom. For convenience, these systems can be denoted as MnCl_3_-T_C_-*N*_z_-zSiCNR-eSi or MnCl_3_-T_C_-*N*_a_-aSiCNR-edge.

Our computed results reveal that all of these MnCl_3_-modified zSiCNR and aSiCNR systems with different ribbon widths can also possess considerable negative adsorption energies in the range of −3.788 to −3.915 eV and −1.680 to −1.730 eV, respectively, in which the distances between the Mn atom and the bonded C atom are in the range of 2.179–2.270 Å, as shown in Table 3.

**Table 3:** The relative energies Δ*E* (meV) of different magnetic couplings to the ground state, the total magnetic moment *M*_tot_, the distance *d*_Mn–C_ between the Mn atom in the adsorbed MnCl_3_ and the bonded C atom, the adsorption energy *E*_ad_, the electronic property and the amount of the electron transfer from SiCNR to MnCl_3_ for the modified zSiCNR and aSiCNR systems with different widths

Systems	Δ*E* (meV)			*M*_tot_ (*μ*_B_)	*d*_Mn–C_ (Å)	*E*_ad_ (eV)	Electronic property	The gap in the minority channel (eV)	The charge of the SiCNR|*e*|
	NM	FM	FM-1						
MnCl_3_-T_C_-6-zSiCNR-eSi	—	24.0	0.0	4.00	2.256	−3.788	Half-metallicity	0.516	0.849
MnCl_3_-T_C_-10-zSiCNR-eSi	—	7.3	0.0	6.00	2.270	−3.836	Half-metallicity	0.469	0.864
MnCl_3_-T_C_-12-zSiCNR-eSi	—	11.5	0.0	6.00	2.262	−3.915	Half-metallicity	0.520	0.899
MnCl_3_-T_C_-9-aSiCNR-edge	1.991	0.0	—	4.00	2.188	−1.730	SGS	—	0.779
MnCl_3_-T_C_-11-aSiCNR-edge	1.991	0.0	—	4.00	2.188	−1.680	SGS	—	0.763
MnCl_3_-T_C_-15-aSiCNR-edge	1.958	0.0	—	4.00	2.179	−1.683	SGS	—	0.742

**Fig. 6:**
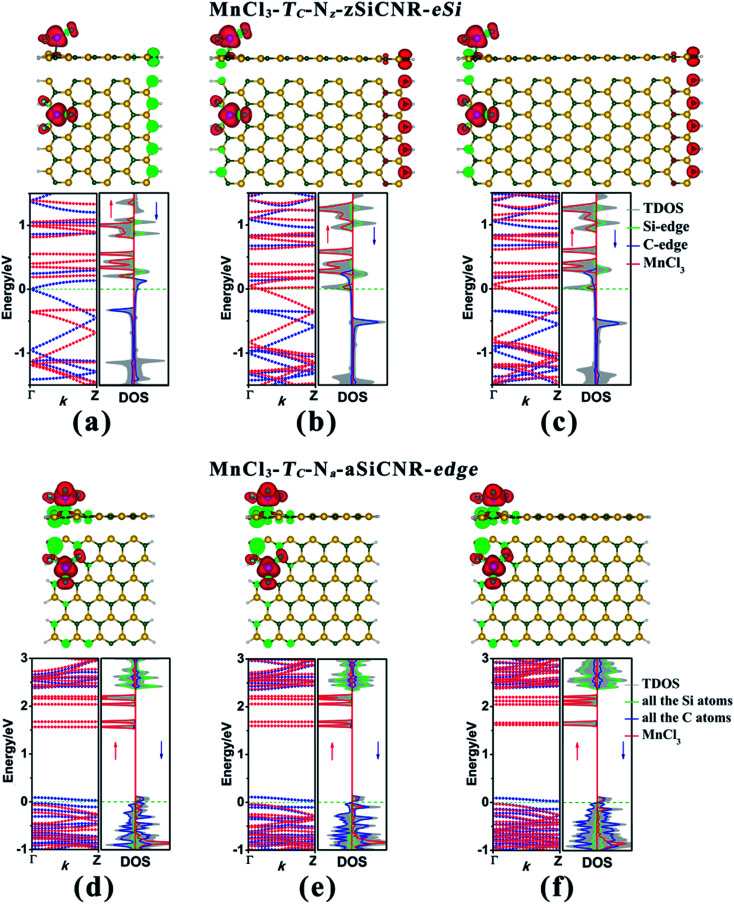
The geometry, electron spin density distribution, band structure and corresponding DOS for the different widths of MnCl_3_-modified SiCNR systems at the ribbon edge, namely, (a) MnCl_3_-T_C_-6-zSiCNR-eSi, (b) MnCl_3_-T_C_-10-zSiCNR-eSi, (c) MnCl_3_-T_C_-12-zSiCNR-eSi, (d) MnCl_3_-T_C_-9-aSiCNR-edge, (e) MnCl_3_-T_C_-11-aSiCNR-edge and (f) MnCl_3_-T_C_-15-aSiCNR-edge. The red and green colors in the electron spin density distribution represent the spin-up and spin-down orientations of the unpaired electrons, respectively. The red and blue dotted lines in the band structures denote the spin-up (↑) and spin-down (↓) channels, respectively. The Fermi-level is set as zero and is indicated by the green dotted line.

As illustrated in Table 3 and Fig. 6, we can find that when the ribbon width *N*_z_ is narrowed from 8 to 6, the half-metallic behavior with the ferromagnetic state (FM-1) can be maintained in the MnCl_3_-modified zSiCNR system at the Si edge, in which the total magnetic moment *M*_tot_ is as large as 4.00 *μ*_B_. The half-metallicity in MnCl_3_-T_C_-6-zSiCNR-eSi can be mainly dominated by the edge C atoms in the spin-down channel across the Fermi level (Fig. 6a). Conversely, when widening the ribbon width *N*_z_ to 10 and even 12, the half-metallic behavior with the ferromagnetic state (FM-1, *M*_tot_ = 6.00 *μ*_B_) can be still sustained in the MnCl_3_-modified zSiCNR systems at the Si edge. The computed DOS results reveal that the half-metallicities in MnCl_3_-T_C_-10-zSiCNR-eSi and MnCl_3_-T_C_-12-zSiCNR-eSi can be uniformly determined by the spin-up states crossing the Fermi level from the edge Si/C atoms and MnCl_3_ (Fig. 6b and c). It is worth mentioning that all the half-metallic behaviors in these three configurations are also rather robust, where the semiconducting spin gap of the minority channel is considerably large with a range of 0.469–0.520 eV (Table 3 and Fig. 6).

Similarly, when narrowing or widening the ribbon width *N*_a_ from 13 to 9, 11 and 15, the SGS behavior with FM ground state (*M*_tot_ = 4.00 *μ*_B_) can be kept in the MnCl_3_-modified aSiCNR systems at the ribbon edge. The computed DOS results reveal that for all of these three systems, both the top valence band from C atoms and the bottom conduction band from the C atoms and MnCl_3_ are uniformly in contact with each other at the Fermi-level, resulting in SGS behavior, as illustrated in Fig. 6d–f.

**Fig. 7:**
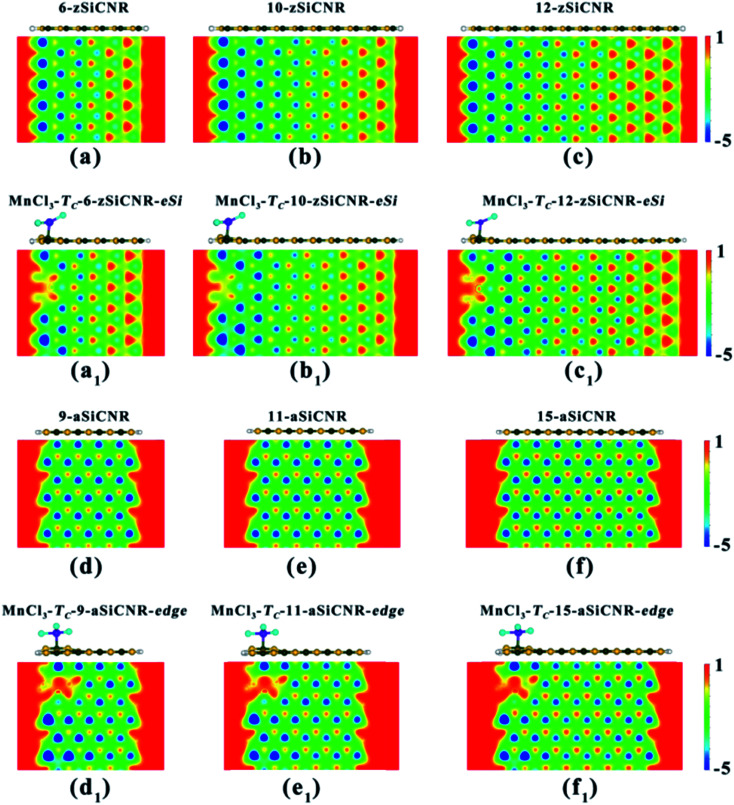
The side view of the geometrical structure and the distribution of electrostatic potential in the nanoribbon plane for the pristine SiCNRs with different widths and the corresponding MnCl_3_-modified SiCNR systems at the edge: (a) pristine 6-zSiCNR, (b) pristine 10-zSiCNR, (c) pristine 12-zSiCNR, (a_1_) MnCl_3_-T_C_-6-zSiCNR-eSi, (b_1_) MnCl_3_-T_C_-10-zSiCNR-eSi, (c_1_) MnCl_3_-T_C_-12-zSiCNR-eSi, (d) pristine 9-aSiCNR, (e) pristine 11-aSiCNR, (f) pristine 15-aSiCNR, (d_1_) MnCl_3_-T_C_-9-aSiCNR-edge, (e_1_) MnCl_3_-T_C_-11-aSiCNR-edge and (f_1_) MnCl_3_-T_C_-15-aSiCNR-edge.

Obviously, independent of the ribbon width, adsorbing the superhalogen MnCl_3_ can effectively engineer band structures of zigzag- and armchair-edged SiCNR systems, owing to the resulting increase of electrostatic potential in the ribbon plane (Fig. 7). The robust half-metallic and SGS behaviors can be maintained for different widths of MnCl_3_-modified zSiCNR and aSiCNR systems at the edge, respectively. It is worth mentioning that we have also doubled the above used supercells to consider the relative energies between the parallel and antiparallel couplings of two neighboring MnCl_3_ sites for the modified SiCNR systems, by sampling the representative systems including MnCl_3_-T_C_-6-zSiCNR-eSi, MnCl_3_-T_C_-8-zSiCNR-eSi, MnCl_3_-T_C_-8-zSiCNR-center, MnCl_3_-T_C_-8-zSiCNR-eC, as well as MnCl_3_-T_C_-9-aSiCNR-edge, MnCl_3_-T_C_-11-aSiCNR-edge, MnCl_3_-T_C_-13-aSiCNR-edge and MnCl_3_-T_C_-13-aSiCNR-center. The computed results reveal that all of these configurations with parallel coupling are lower in energy than the corresponding ones with antiparallel coupling (Table S1†), indicating that parallel coupling between two neighboring MnCl_3_ units can be more stable. Therefore, all the computed results based on the supercell containing one MnCl_3_ should be reliable.

## 4 Conclusions

On the basis of first-principles computations, we have performed a detailed theoretical study on the structures, and the electronic and magnetic properties of modified zSiCNR and aSiCNR systems by adsorbing the magnetic superhalogen MnCl_3_, which has a strong electron-withdrawing ability and a large intrinsic magnetic moment. The following intriguing findings are reported:

(1) when adsorbing MnCl_3_ at the ribbon center, magnetism can be introduced in the substrate SiCNR, and simultaneously an electron transfer process can be induced from SiCNR to MnCl_3_, leading to an evident increase of electrostatic potential in the ribbon plane, like applying an electric field. As a result, the magnetic degeneracy of pristine zSiCNR can be broken and the strong ferromagnetic metallicity can be observed, while the nonmagnetic semiconductor aSiCNR can be transformed to exhibit a robust ferromagnetic half-metallicity.

(2) When moving MnCl_3_ from the center to the edge, the ferromagnetic metallicity can be changed to a robust ferromagnetic half-metallicity in the modified zSiCNR system, while the ferromagnetic half-metallicity can be converted into ferromagnetic SGS behavior in the modified aSiCNR system. Clearly, the adsorption site of MnCl_3_ has an important effect on the electronic and magnetic behaviors in the joint systems.

(3) Independent of the ribbon width, the robust ferromagnetic half-metallic or SGS behavior can be sustained in the MnCl_3_-modified zSiCNR and aSiCNR systems at the edge, respectively.

(4) All of these new MnCl_3_-modified SiCNR systems can exhibit large adsorption energies in the range of −1.430 to −3.915 eV, indicating they possess high structure stabilities.

Undoubtedly, adsorbing the magnetic superhalogen MnCl_3_ can be a new strategy to effectively modulate the electronic and magnetic properties of zSiCNR and aSiCNR systems. These intriguing findings can be advantageous for promoting the practical application of excellent SiC-based nanomaterials in spintronics and multifunctional nanodevices.

## Conflicts of interest

There are no conflicts to declare.

## Supplementary Material

RA-008-C8RA01632A-s001
