# Supplementary material for: Adsorbing the magnetic superhalogen MnCl3 to realize intriguing half-metallic and spin-gapless-semiconducting behavior in zigzag or armchair SiC nanoribbon
Source: RSC Adv. 2018 Apr 11;8(24):13167–77. doi: 10.1039/c8ra01632a (PMC9079843; doi:10.1039/c8ra01632a)
Supplement: RA-008-C8RA01632A-s001 [file RA-008-C8RA01632A-s001.pdf]

Electronic Supplementary Material (ESI) for RSC Advances.
This journal is © The Royal Society of Chemistry 2018

Electronic Supplementary Information

Adsorbing the Magnetic Superhalogen $MnCl_3$ to Realize the Intriguing Half-metallicity and Spin-Gapless-Semiconductor in Zigzag or Armchair SiC Nanoribbon

Hui Li, Guangtao Yu*, Zengsong Zhang, Yanfeng Ma, Xuri Huang, Wei Chen*

Laboratory of Theoretical and Computational Chemistry, Institute of Theoretical Chemistry, International Joint Research Laboratory of Nano-Micro Architecture Chemistry, Jilin University, Changchun 130023, People's Republic of China

*To whom correspondence should be addressed. Email: yugt@jlu.edu.cn (G.Y.), w_chen@jlu.edu.cn (W.C.)

(Ⅰ) The computational test on the modified 8-zSiCNR and 13-aSiCNR systems with superhalogen $MnCl_3$ at the center

When adsorbing the superhalogen $MnCl_3$ at the ribbon center for 8-zSiCNR and 13-aSiCNR, we consider all four possible adsorption sites for the Mn atom in $MnCl_3$ including the top site of C atom (T_C), top site of Si atom (T_{Si}), bridge site over Si-C bond (*B*) and hollow site of SiC hexagon ring (*H*). The computed results reveal that the modified zSiCNR or aSiCNR configuration with the $MnCl_3$ at the bridge site of ribbon center cannot be obtained. Consequently, we can respectively obtain three conformations for the $MnCl_3$-modified 8-zSiCNR and 13-aSiCNR systems at the center (Figures S1 and S2). By comparison, we can find that adsorbing the $MnCl_3$ at the top of C atom (T_C) can obtain the most energetically stable configurations for the modified zSiCNR and aSiCNR systems (Figures S1 and S2), and they have been correspondingly named as $MnCl_3$-T_C-8-zSiCNR-*center* and $MnCl_3$-T_C-13-aSiCNR-*center* in the main text.

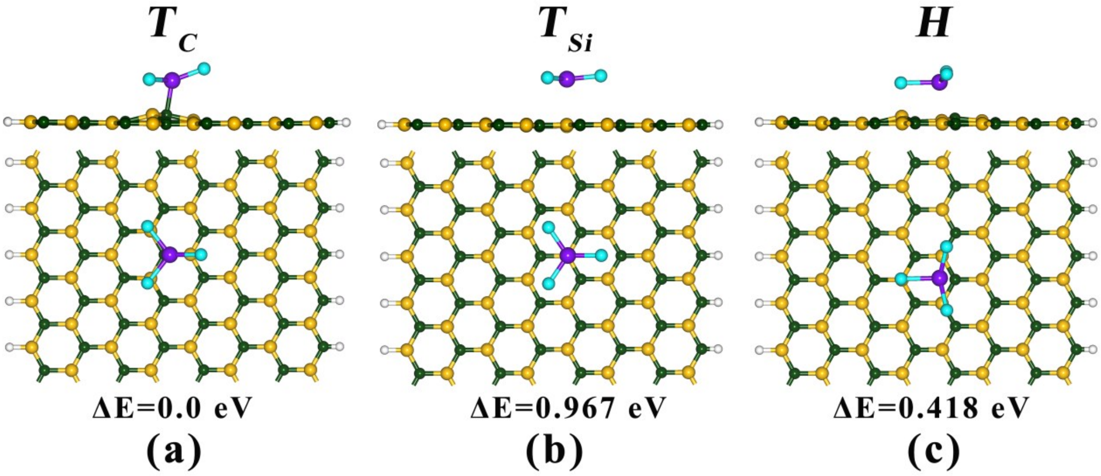

Figure S1 The side view and top view of modified 8-zSiCNR with the superhalogen $MnCl_3$ at the ribbon center, and the relative energy ΔE (eV) between the $MnCl_3$-modified 8-zSiCNR systems at three different adsorption sites including (a) T_C, (b) T_{Si} and (c) *H*.

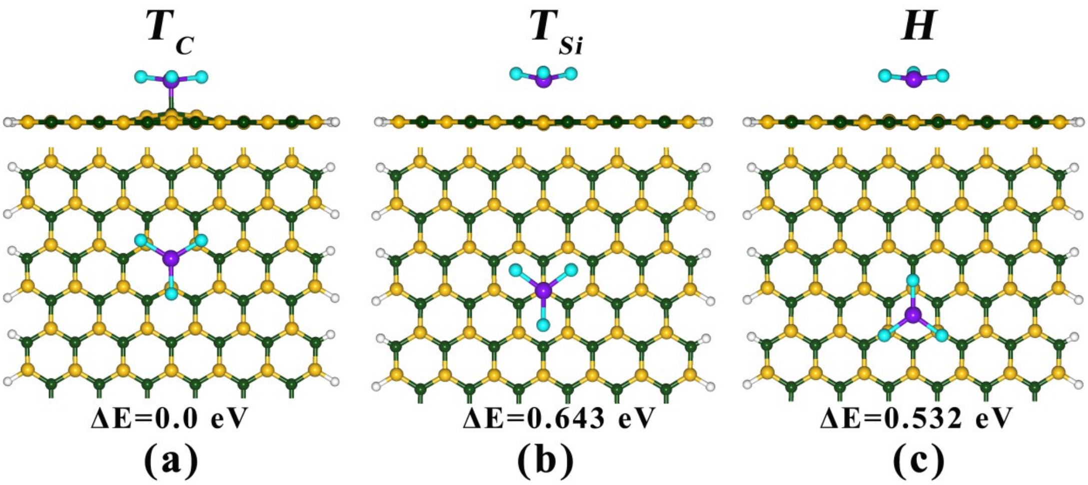

Figure S2 The side view and top view of modified 13-aSiCNR with the superhalogen $MnCl_3$ at the ribbon center, and the relative energy ΔE (eV) between the $MnCl_3$-modified 13-aSiCNR systems at three different adsorption sites including (a) T_C, (b) T_{Si} and (c) H.

(Ⅱ) The computational test on the $MnCl_3$-modifed SiCNR systems by using the double supercell

Table S1. The relative energies ΔE (meV) between the parallel and antiparallel couplings of two neighboring $MnCl_3$ for the modified SiCNR systems. Note that these modified SiCNR systems with two $MnCl_3$ in the supercell can be denoted by adding (double) into the names of corresponding ones with one $MnCl_3$.

Systems	ΔE (meV)	
	Parallel	Antiparallel
$MnCl_3$-T_C-6-zSiCNR-*eSi*(double)	0.0	1.5
$MnCl_3$-T_C-8-zSiCNR-*eSi*(double)	0.0	4.3
$MnCl_3$-T_C-8-zSiCNR-*center*(double)	0.0	1.4
$MnCl_3$-T_C-8-zSiCNR-*eC*(double)	0.0	65.4
$MnCl_3$-T_C-9-aSiCNR-*edge*(double)	0.0	3.2
$MnCl_3$-T_C-11-aSiCNR-*edge*(double)	0.0	1.2
$MnCl_3$-T_C-13-aSiCNR-*edge*(double)	0.0	3.5
$MnCl_3$-T_C-13-aSiCNR-*center*(double)	0.0	3.3
